# The Oriental hornet, *Vespa orientalis* Linnaeus, 1771 (Hymenoptera, Vespidae): diagnosis, potential distribution, and geometric morphometrics across its natural distribution range

**DOI:** 10.3389/finsc.2024.1384598

**Published:** 2024-10-29

**Authors:** Allan H. Smith-Pardo, Mariano Altamiranda-Saavedra, P. David Polly

**Affiliations:** ^1^ United States Department of Agriculture (USDA), Animal and Plant Health Inspection Service (APHIS), Plant Protection and Quarantine (PPQ), Science and Technology (S&T), Sacramento, CA, United States; ^2^ Grupo de Investigación Bioforense, Tecnológico de Antioquia Institución Universitaria, Medellín, Colombia; ^3^ Earth and Atmospheric Sciences, Biology and Anthropology, Indiana University, Bloomington, IN, United States; ^4^ Geosciences & Geography, University of Helsinki, Helsinki, Finland

**Keywords:** insect biology, invasive species, niche modeling, shape variation, wasps

## Abstract

We present a short review of the biology, diagnostic characteristics, and invasiveness of the Oriental hornet, *Vespa orientalis*. We also performed an analysis of the shape of the forewings (geometric morphometrics) of different geographic groups along their native distribution and their potential geographical distribution using the MaxEnt entropy modeling. Our results show a wide potential expansion range of the species, including an increase in environmentally suitable areas in Europe, Asia, and Africa but more especially the Western Hemisphere, where the species was recently introduced. The geometric morphometric analysis of the forewings shows that there are three different morphogroups: one distributed along the Mediterranean coast of Europe and the Middle East (MEDI), another along the Arabian Peninsula and Western Asia but excluding the Mediterranean coast (MEAS), and one more in northern Africa north of the Sahara and south of the Mediterranean coast (AFRI), all of which show differences in their potential distribution as a result of the pressure from the different environments and which will also determine the capacity of the different morphogroups to successfully invade new habitats.

## Introduction

Hornets (genus *Vespa*) are eusocial wasps that belong to the family Vespidae, subfamily Vespinae. Currently, there are 22 recognized species of hornets (1, 2). Most species of *Vespa* are native to the tropical parts of Asia, although some species such as *Vespa crabro* and *Vespa orientalis* reach the Asian Palearctic and Europe and northern Africa (2–4).

Hornets are omnivorous, feeding on various resources from sap and fruits to invertebrates and even carrion from larger animals. Most species are opportunistic (except for *Vespa dybowskii* André, 1884, a social parasite of other species of *Vespa*—2, 5) and will feed where resources are abundant and localized, which is part of the reason why some of the species in the genus can become pests of honeybees or attack other social Hymenoptera. The biology of the species in *Vespa* is also diverse. Some species are predators, while others are social parasites of other species in the same genus. Their nesting behavior is also diverse, with some species nesting in ground burrows or above ground in hollow trees, rock crevices, or human-made structures (4, 6, 7).

Some of the species of the genus *Vespa* have also been introduced into regions outside their natural range. These include *V. crabro* in Eastern USA and Canada (Bequaert 1932, 8); *Vespa velutina* in parts of Europe, South Korea (9), and the State of Georgia (USA); *Vespa mandarinia* in the state of Washington, USA, and British Columbia, Canada (10, 11); *Vespa tropica* in the island of Guam (12); and *V. orientalis* in parts of Europe, Africa, and Chile (13–18, among others).

The taxonomy, biology, and nesting biology of the Oriental hornet, *V. orientalis*, was initially studied by Archer (1), while Werenkraut et al. (19) studied their potential distribution and potential treatment after the species was reported by Rios et al. (17) as introduced and established in Chile.

Nests of *V. orientalis* can be found underground or above ground in cracks in rocks or walls or under roofs of human-constructed structures (1). *V. orientalis*, like other congeneric species, is highly social. Colonies start with a mated queen emerging from overwintering with a colony cycle that goes (in North Africa and the Middle East) from mid-late April to the end of November in some cases, although sometimes a few workers and males may persist into December. Mating flights take place from October into November, with the fertilized queens entering over-wintering sites by the end of November (1, 20).

The natural distribution of *V. orientalis* includes the north of Africa, southern Europe, and the Mediterranean region (the Middle East, including the Arabian Peninsula and Southwestern Asia) (1) (**Supplementary Material 8**).

The potential of *V. orientalis* as an invasive species is demonstrated by its current distribution compared to its natural range. There are, however, some questions regarding the necessary conditions for the establishment of this species, as it has been reported in countries such as Madagascar (21, 22) and Mexico (13) but has not been recorded for many years after the initial reports. Likely, it did not get established. Archer (1) reported a specimen from the province of Fujian in SW China, but he assumed that it was transported there accidentally. More recently, Otis et al. (23) evaluated the invasive potential of this and other species of hornets, genus *Vespa*.

Oriental hornets have also been dubiously recorded for Brazil and Guiana by Guiglia (24) and “sporadically” intercepted in Belgium and the United Kingdom according to Kimsey et al. (25). The most recent reports of potentially successful introductions include Chile between 2018 and 2020 (17), north of Italy between 2018 and 2019 (Trieste) where it was also found nesting in urban settings (26), and Romania between 2019 and 2021 also in an urban setting, Bucharest (18).

The Oriental hornet has also been associated with honeybee pathogens and has been considered a potential vector of honeybee pathogens and a threat to public health (27, 28).

In terms of its appearance and morphology, the Oriental hornet is similar to some other species in the genus *Vespa* by a combination of the following characteristics: the presence of the pronotal and pretegular carinae, a large vertex (=distance from the posterior ocellus to the posterior margin of the vertex is more than twice the distance between the posterior ocelli and the compound eyes) and, in the forewings, a prestigma that is much longer than the stigma (=three times or more the length of the pterostigma); illustrations of these and other diagnostic characteristics for the genus can be seen in Smith-Pardo et al. (1).

In this work, we look at the shape variation of the wings of museum specimens of *V. orientalis* and the potential distribution of the different groups obtained based on wing shape. In order to do this, we present a geometric morphometric analysis of the forewings of three resulting morphogroups of *V. orientalis* from previous analyses and distributed along the circum-Mediterranean region to determine whether the source morphogroup can be differentiated based on the shape of their forewings and whether individuals can be accurately provenanced to one of these regions based on the wing shape criterion.

We also conduct an ecological niche model analysis to estimate the climate envelopes for each morphogroup to develop hypotheses for which areas of the world are likely to provide suitable habitats. Specifically, we are interested in which morphogroups may be the sources of recent invasions of the Western Hemisphere, such as the one newly established in Chile in the early 2020s, older isolated records in Mexico and Brazil, and interceptions in the continental USA on military aircraft from the Middle East.

## Materials and methods

### Identification of *V. orientalis* based on its external morphology

**Figure 1:**
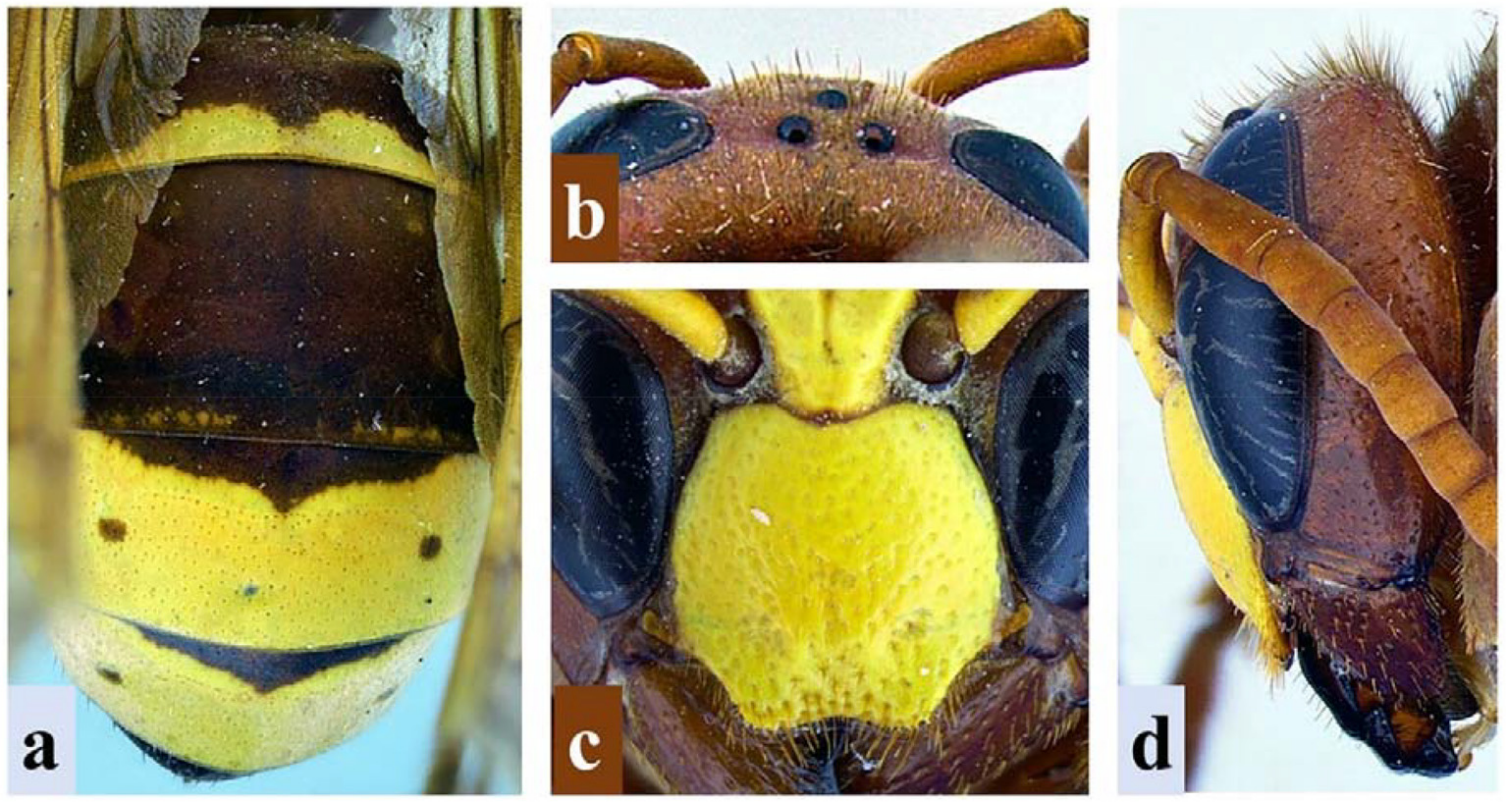
Diagnostic characteristics of *Vespa orientalis* L. **(A)** Dorsal view of metasoma showing the coloration patterns in T1–T4. **(B)** Vertex of the head showing inter-ocellar distance. **(C)** Close-up of clypeus showing clypeal margins. **(D)** Lateral view of the head showing the width of the gena and compound eye.

The subject of this study, *V. orientalis*, can be differentiated from other species in the genus by its coloration pattern [=metasoma reddish to dark brown with terga three (T3) and four (T4) mostly yellow with a basal reddish-brown band that extends medially and two small lateral spots], posterior ocelli that are closer to each other than to the compound eyes, the lack of a medial clypeal “tooth” between the two broadly rounded lateral projections, the genae that is as wide or slightly wider (no more than 1.5 times) than the width of the compound eye in lateral view, and the presence of a complete pretegular carina (**Figure 1**) (1).

The Oriental hornet is in fact, highly variable in its coloration patterns. According to Ascher (1998), there are six color forms of the species that were described as subspecies originally: *V. orientalis jurinei* de Saussure, 1853; *V. orientalis aegyptica* André, 1884; *V. orientalis zavattarii* Guiglia & Capra, 1933; *V. orientalis somalica* Giordahi-Soika, 1934; and *V. orientalis arabica* Giordani-Soika, 1957. Smith-Pardo et al. (1) presented a list of synonyms that included 11 names (junior synonyms) from subspecies to species.

There are no studies in the morphometry of the species, in particular on the geometric morphometrics of the forewings (a structure normally used in the studies of shape in hornets) of all color variations of the species.

### Specimens studied

Information for the morphometric analysis presented here was obtained using images of the right forewing of specimens housed at the American Museum of Natural History, New York (AMNH, James Carpenter and Christine Lebeau), the Bohart Museum of Entomology, University of California, Davis (BMEC, Lynn Kimsey and Steve Heydon), and borrowed images of specimens from other collections in Europe, Asia, and Africa provided by Dr. Adrien Perrard and which were part of work of Perrard et al. (29). The sample size for this study, particularly for the geometric morphometric data gathering, therefore, was limited by the number of specimens correctly identified and well preserved in collections of Entomology around the world with a good representation of specimens of the species *V. orientalis*.

Preliminary results from Smith-Pardo (unpublished data) have shown that there are three morphogroups of *V. orientalis* along its natural distribution range. Therefore, we grouped the specimens in this study into three different geographical regions: northern Africa, the Middle East (including countries in the Arabian Peninsula), and the Mediterranean (including Turkey but excluding countries listed in the Middle East). A list of specimens and their collecting locations is shown in **Supplementary Material 1**.

### Geometric morphometrics of *V. orientalis*

**Figure 2:**
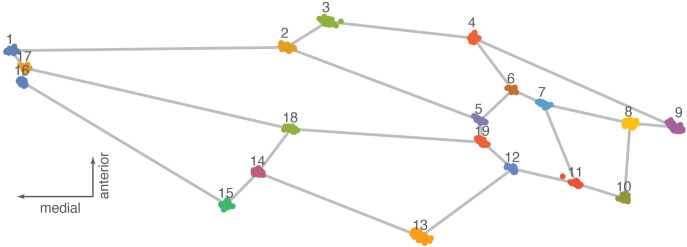
Procrustes fit showing landmarks with data points for all the specimens of *Vespa orientalis* used in this study.

For the geometric morphometric analyses (30), we used the software MorphoJ v. 1.08.01 (31, 32) to perform a Procrustes fit; we also looked at the outliers, generated a covariance matrix, and obtained a principal component analysis of the data and a Procrustes ANOVA for comparisons of the landmark coordinates of the specimens in the three regions; finally, we compared the results using a canonical variate analysis. The “Procrustes ANOVA” test, as it is called in the MorphoJ software package, is technically a multivariate analysis of variance (MANOVA) because it utilizes all of the landmark coordinates in testing for group differences. The analyses were cross-checked using both PAST v. 4.15 (33) and Geometric Morphometrics for Mathematica v. 12.5 (34). **Figure 2** shows the landmarks used for the forewings (after Perrard et al. 29). Images of the forewings were landmarked with the program tpsDig 232 (35) and using the same 19 landmarks used by Perrard et al. (29) as a reference. Landmark coordinates were first edited in MS Word, opened in the text editor, and saved as a*.txt* file to be used as the database for the geometric morphometric (GMM) analysis. Landmark coordinates for the specimens used in this analysis are available in **Supplementary Material 2**. Procrustes analysis is a necessary step to superimpose landmark data by scaling, translating, and rotating them to place them in a common coordinate system in which their shape distances are minimized (36, 37). Principal component analysis (PCA) shows patterns of shape similarity and difference and provides shape variables (PC scores) for further statistical analysis (38, 39). Procrustes ANOVA tests whether the forewing shape of the three morphogroups is statistically different given our samples (40).

Canonical variate analysis (CVA) and its associated discriminant function analysis (DFA) are used to find the combination of variables that best distinguish the groups for use in classification (37, 39, 41). We used CVA to find the forewing shape that best diagnoses the differences between forewing shapes in the three geographic groups and then used the canonical variate loadings as discriminant functions to conduct a leave-one-out cross-validation in which the forewing shape is used to classify individual hornets to determine the frequency with which they are correctly classified. Because CVA weights input variables by their ability to discriminate between known groups, it is particularly prone to finding spurious differences between groups when the number of variables greatly exceeds the number of groups or number of objects in the analysis, as is often the case with geometric morphometric data (e.g., 42, 43). One solution to this over-parameterization is to reduce the number of variables using PCA and discard those components with little variance. For our CVA, we used the scores of the first nine PCs, which collectively explained 90% of the shape variance in our data set but still allowed for three variables per geographic group for discriminatory power.

### Modeling potential distribution of *V. orientalis*

Ecological niche modeling parameters were estimated using the R Program ENMeval package (44) for each of the morphological groupings identified with geometric morphometric analysis to model each group’s potential geographic range.

We gathered data from the Global Biodiversity Information Facility database (GBIF.org, 2023; https://www.gbif.org/) regarding the documented distribution of *V. orientalis*. Following the proposed morphometric criteria, we classified these records into three distinct geographic regions—Africa (AFRI), the Middle East (MEAS), and the Mediterranean (MEDI)—as well as all the data as a single morphogroup. To ensure data quality, we eliminated duplicate entries and records with inconsistent georeferencing, such as those falling beyond national borders or within marine areas. The defined “calibration area”, according to Rojas-Soto et al. (45), employed a buffer of 100 km around existing specimen records. Finally, to model the potential distribution, we used 320 records for MEDI, 164 for MEAS, and 17 for AFRI; the last one had a much smaller set than the other two morphogroups due to a smaller number of specimens collected and observed in this part of the world that is less studied and which fauna of invertebrates are less known than in the other two regions. Different numbers of data may result in conclusions that can be driven from the results. Still, they also represent an opportunity to call on the scientific community’s attention to study this area as a potential source of invasive species.

To characterize the fundamental niche of these geographical morphogroups, we used environmental data from WorldClim, version 2.0 (spatial resolution = 30 arcseconds, approximately 1 km) (46). To mitigate environmental layer collinearity and select environmental variables set, we conducted Pearson’s correlation analysis for each morphogroup using the R package “ntbox” (47). Variables exhibiting correlation values exceeding 0.8 were excluded from the final set by morphogroups (48) (**Table 1**).

**Table 1:** Set of environmental variables used for construction of the potential distribution model and ecological niche model for *Vespa orientalis* morphogroups.

Environmental variables	Code	Morphogroup MEAS	Morphogroup MEDI	Morphogroup AFRI
Mean annual air temperature	Bio1	X	X	X
Mean diurnal air temperature range	Bio2		X	
Isothermality	Bio3	X		
Temperature seasonality	Bio4		X	X
Mean daily maximum air temperature air of the warmest month	Bio5	X		
Mean daily maximum air temperature air of the coldest month	Bio6	X	X	X
Annual range of air temperature	Bio7	X	X	X
Mean daily mean air temperature air of the wettest quarter	Bio8			
Mean daily mean air temperature air of the driest quarter	Bio9			
Mean daily mean air temperature air of the warmest quarter	Bio10	X		
Mean daily mean air temperature air of the coldest quarter	Bio11	X		X
Annual precipitation amount	Bio12		X	X
Precipitation amount of the wettest month	Bio13	X	X	X
Precipitation amount of the driest month	Bio14	X	X	
Precipitation seasonality	Bio15			X
Mean monthly precipitation amount of the wettest quarter	Bio16		X	
Mean monthly precipitation amount of the driest quarter	Bio17			
Mean monthly precipitation amount of the warmest quarter	Bio18			
Mean monthly precipitation amount of the coldest quarter	Bio19			X

MEDI, Mediterranean; AFRI, Africa; MEAS, Middle East.

We assessed the ecological niche model adequacy by evaluating its ability to recover the current geographical distribution using MaxEnt 3.3.3k (49). To determine the optimal parameterization of suitability estimates in the calibration region, we tested various settings using the ENMeval package within the R program. This package offers an automated approach for executing MaxEnt models over a user-defined range of regularization multiplier (RM) values and feature combinations (FCs). We specified an RM range from 0.5 to 4.0 in 0.5 increments, along with three FCs, namely, linear (L); linear and quadratic (LQ); linear, quadratic, and product (LQP); linear, quadratic, product, and threshold (LQPT); and linear, quadratic, product, threshold, and hinge (LQPTH), leading to 45 potential feature and regularization multiplier combinations (44). We used output format (row), number of replicates (10), testing data percentage (25%), validation type (bootstrapping), maximum iteration count (5,000), convergence threshold (0.00001), and maximum background points (10,000). No clamping or extrapolation was applied consistently in all MaxEnt runs. Using the lowest training presence threshold (LTPT) method (50) with a permissible omission error rate of E = 5%, we transformed the continuous potential distribution map into a binary map, representing the environmental suitability areas in geographic space. Different feature combinations impact the results by adding other responses to bioclimatic variables. Therefore, we used the ENMeval package, which executes a series of models across a user-defined range of settings (i.e., combinations of feature classes and regularization multiplier values). Finally, it provides six evaluation metrics to characterize model performance and help choose the best final model in the calibration area. The final model in the global projection area could not be statistically evaluated due to the lack of presence records that met the minimum independence requirement.

### Assessing niche overlap

To assess niche overlap among the three geographic morphogroups, we conducted a niche overlap analysis using Niche Analyst 3.0 (51). An environmental space was constructed based on the first three principal components, which encapsulated 90% of the variation in the Worldclim climatic variables. We generated simulations of virtual niches that corresponded to the total occurrence points for each morphogroup and determined the minimum-volume ellipsoid. The Jaccard index (IJ) was used to estimate environmental overlap among species (51).

## Results

### Geometric morphometrics

**Figure 3:**
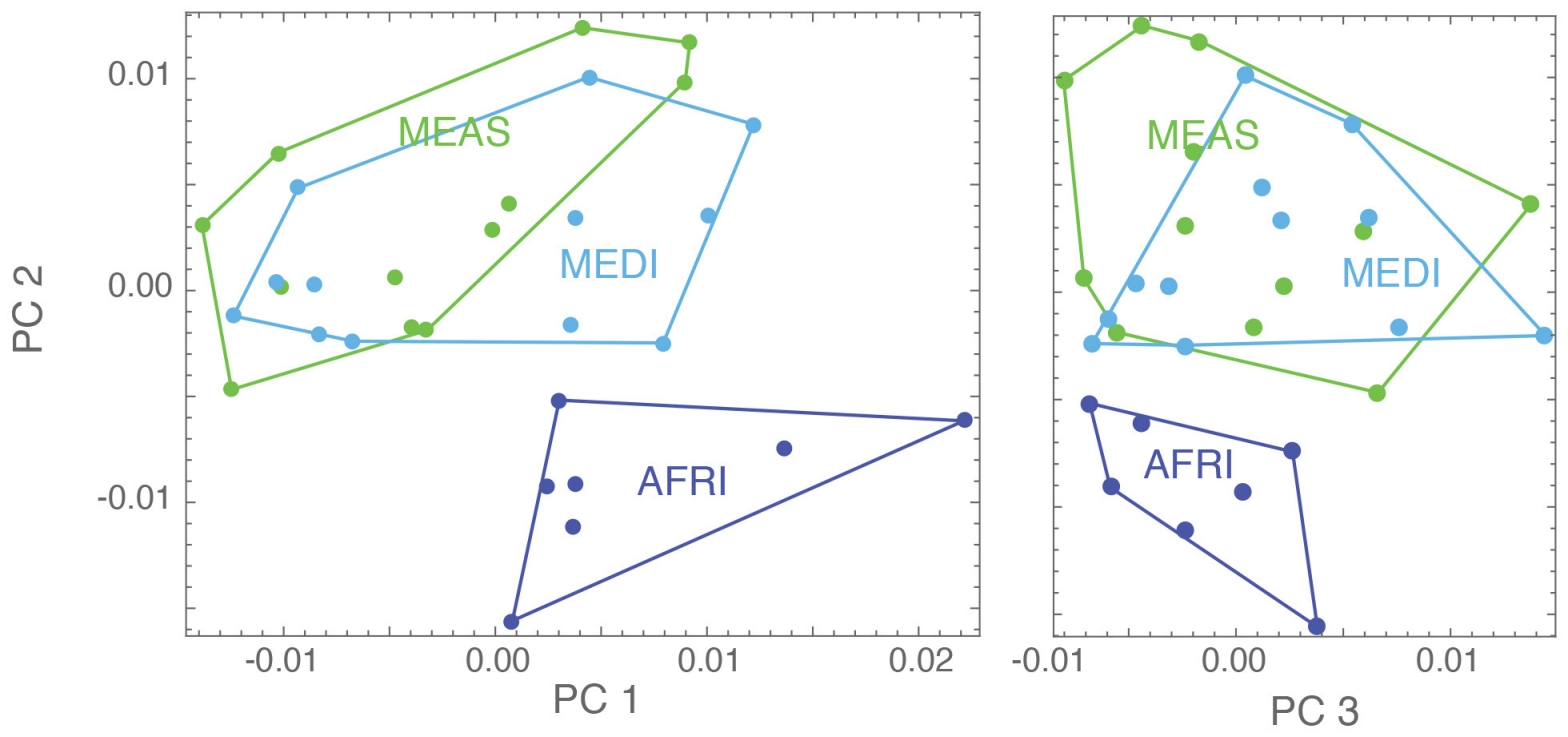
Scores for three of the principal components for the present analysis (PC1, PC2, and PC3): each dot represents a specimen, and its color represents the geographical region it belongs to [dark blue = Africa (AFRI), green = Middle East (MEAS), and light blue = Mediterranean (MEDI)].

**Figure 4:**
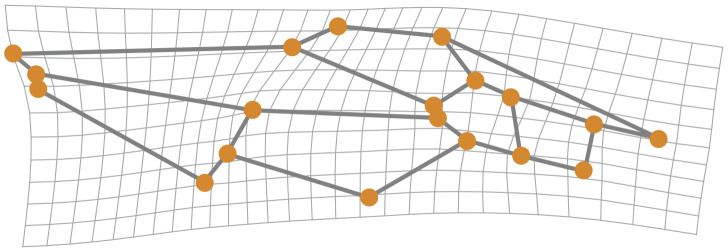
Displacement at each landmark in the positive direction along principal component 1.

Analysis of shape variation in the forewings of these samples shows that the African (AFRI) morphogroup is more differentiated than the other two morphogroups. The variation at each landmark after Procrustes fit is shown in **Figure 2**. Some landmarks have more variation (i.e., landmarks 3, 9, 11, and 13) than others (i.e., landmarks 1, 4, and 12), which indicates that these locations are the key features involved in the differentiation. The first three dimensions of PCA morphospace, the dimensions of which are based on overall similarity and difference in forewing shape, show how that variation separates the groups (**Figure 3**). PC1 accounts for 28.0% of the total forewing shape variation, PC2 accounts for 16.1%, and PC3 accounts for 12.1%. The first nine principal components collectively account for 90% of the shape variation and are used below for canonical variate/discriminant function analysis. The first principal component is associated with elongated distal parts of the forewing and compressed medial parts (**Figure 4**). The morphospace plot shows that the African (AFRI) morphogroup is distinctive from the northern Mediterranean (MEDI) and Middle Eastern (MEAS) morphogroups, which themselves are pretty similar to each other on all three PCs. Procrustes ANOVA shows that there is overall a significant difference among the mean shapes of the morphogroups (*F* = 3.11, *p* = 0.0004), but *post-hoc* pairwise comparisons show that while the northern African morphogroup is significantly different from the two others (AFRI-MEAS *p* < 0.0001; AFRI-MEDI *p* = 0.0008), the north Mediterranean and Middle Eastern groups are not substantially different from each other (MEDI-MEAS *p* = 0.52). These results indicate that the bulk of the variation in forewing shape is due to the differentiation of the northern African group from the Mediterranean and Middle Eastern groups.

**Figure 5:**
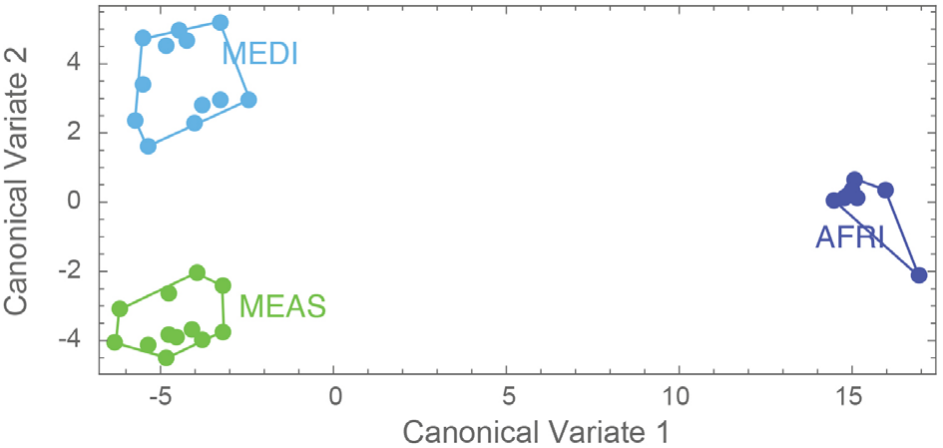
Results of the canonical variate analyses (1 and 2), where dark blue represents morphogroup populations from the African region (AFRI), green morphogroup populations from the Middle East region (MEAS), and light blue morphogroup populations from the Mediterranean region (MEDI).

The aspects of forewing shape distinguish the morphogroups. The first nine principal component shape variables were extracted using CVA. The CVA shows that not only is the AFRI group distinctive, as seen in the PCA, but that smaller but consistent differences in forewing shape distinguish the MEDI and MEAS groups (**Figure 5**). The effectiveness of these differences for distinguishing the morphogroups can be tested by cross-validation DFA using the equations estimated from the CVA. It was found that, overall, 83.9% of individuals were correctly classified. Individuals from the northern African group were classified correctly 100% of the time, consistent with their pronounced differentiation. Middle Eastern and north Mediterranean groups were classified correctly between 75% and 83% of the time (**Table 2**). Thus, even though Procrustes ANOVA did not find a significant difference between the mean forewing shapes of the MEDI and MEAS morphogroups, many individuals from these groups can nevertheless be accurately assigned based on their wing shape, as suggested by their separation in the CVA.

**Table 2:** Confusion matrix from discriminant function analysis showing the ability of forewing shape to correctly classify individuals to morphogroups of *Vespa orientalis* from the three regions of natural distribution.

	AFRI	MEAS	MEDI	Classified correctly	
AFRI	**7**	0	0	7	100%
MEAS	0	9	3	12	75%
MEDI	0	2	10	12	83%
Correct classifications	7	11	13	31	
	100%	82%	77%		

Rows are the known morphogroup, and columns are the group to which they are classified.

AFRI, Africa; MEAS, Middle East; MEDI, Mediterranean.

Additional results of the geometric morphometric analysis are presented in **Supplementary Materials 2**, **3**, **5**.

### Potential distribution

**Figure 6:**
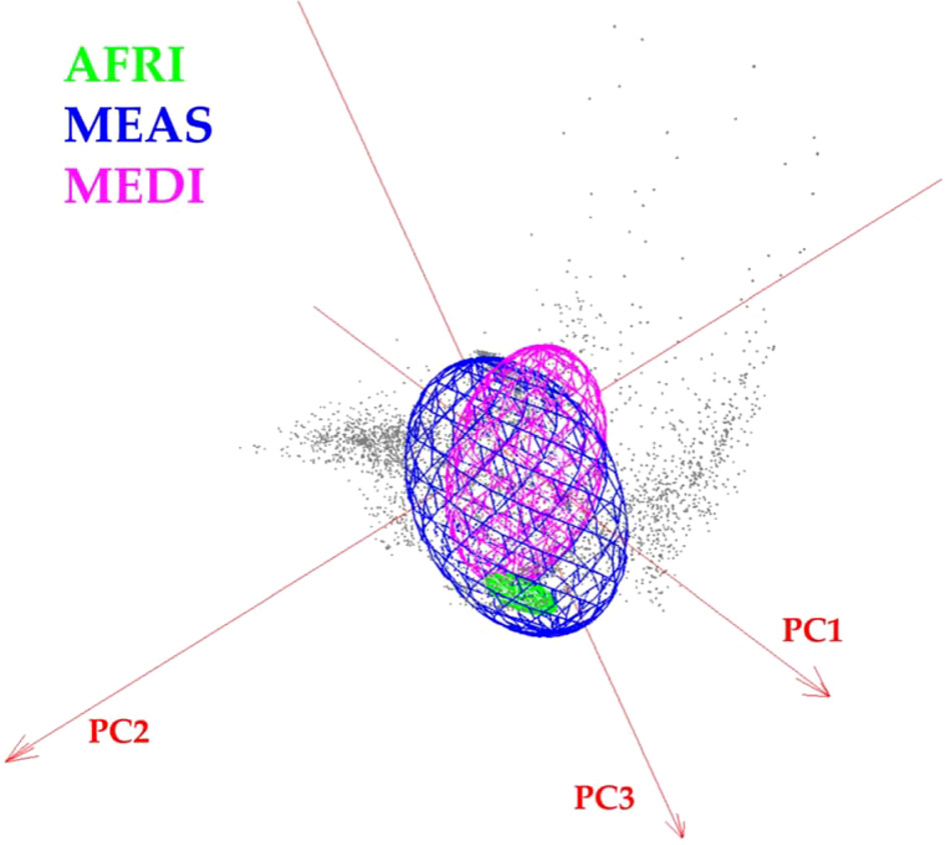
Minimum-volume ellipsoid (MVE) niche estimations for *Vespa orientalis* geographic morphogroups in three-dimensional climate space.

**Figure 7:**
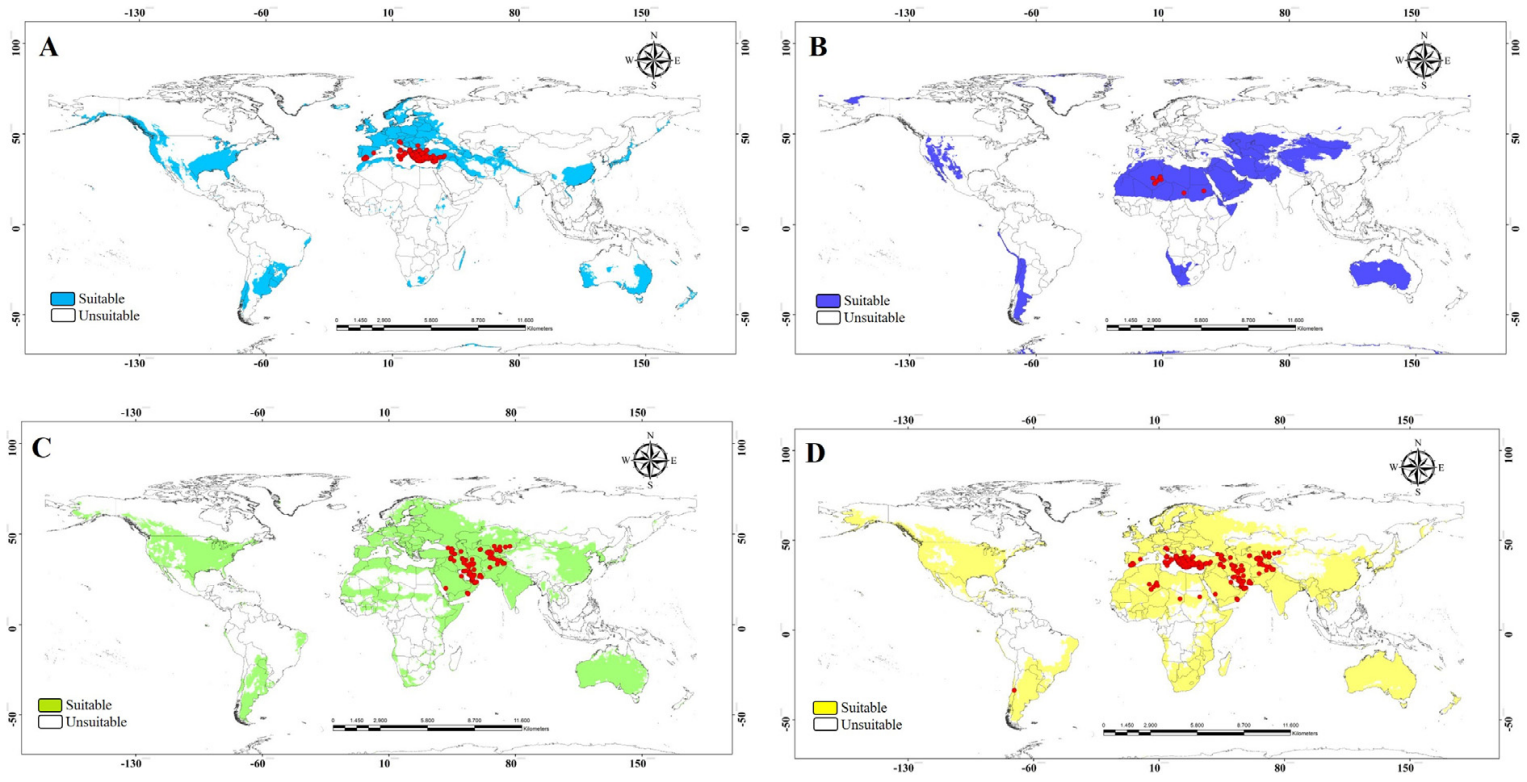
Suitable and unsuitable areas for the potential distribution of *Vespa orientalis*. **(A)** MEDI. **(B)** AFRI. **(C)** MEAS. **(D)** All morphogroups; red circles represent the presence records to model calibration. MEDI, Mediterranean; AFRI, Africa; MEAS, Middle East.

The ecological niche model analysis was used to determine which parts of the world will likely provide suitable habitats for invasive populations of the three morphogroups. This information may help predict high-risk areas for invasion, and conversely, it may help pinpoint the likely source of already established invasive species. Our data were grouped into four treatments (the three morph groups and a combined analysis). Ecological niche model outputs showed heterogeneous patterns across the world (**Figure 6**). The binary models of the potential distribution for the MEDI morphogroup indicated that the area with the highest environmental suitability extends from Europe, a small region of southern South America, and eastern North America (**Figure 7A**). The potential distribution of the AFRI morphogroup showed suitable areas in North Africa and small zones in Asia, as well as in some patches of the Austral region in South America and west of the USA (**Figure 7B**). The map for the morphogroup MEAS has a vast potential distribution in the northern and southern temperate zones of the world (**Figure 7C**). The model that includes records from all morphogroups shows that the wide potential distribution is a combination of the environmentally suitable areas of the three morphogroups (**Figure 7D**). Notably, the models of the three morphogroups predict environmental suitability near or in the record of the species in Chile previously reported by Rios et al. (17), with the presence record only included in the model with all morphogroups.

### Niche overlap

The visualization of the environmental space occupied by the different morphogroups revealed broad overlap among the niches of the three morphogroups and showed that niche breadth was greater for MEAS. The Jaccard index indicated that the environmental overlap between MEAS and MEDI was 0.31, that for AFRI versus MEDI was 0.13, and that for AFRI versus MEAS was 0.0014.

This study shows significant differences in the shape of the forewings among the specimens of *V. orientalis* used and that these differences are related to their distributions. Such differences in shape show that there are at least three groups (morpho groups) associated with their current distributions: an African group (AFRI) that encompasses the specimens around the Sahara Desert south of the Mediterranean coast; a Mediterranean group (MEDI) that includes specimens from the Mediterranean Coast, which is probably the source of the expansion of this species into most of Europe based on the ecological niche distributions; and finally a group from the Middle East (MEDI) that includes the Arabian Peninsula, Western Asia, and possibly some of the populations in northern Europe. It is necessary to clarify this through a comprehensive study on the population genetics of *V. orientalis* found across Europe, which is outside the scope of this work.

## Discussion and conclusions

The results of the study of shape indicate that the northern African (AFRI) morphogroup is strongly differentiated from the others but with substantially less differentiation between the north of Mediterranean (MEDI) and Middle Eastern (MEAS) groups. Morphogroups of *V. orientalis* from countries at the edges of two regions (i.e., Egypt in North Africa and from the Arabian Peninsula, listed as part of the Middle East, or Turkey as part of the Mediterranean and part of the Middle East group) are different in shape of their wings. They are still isolated from one another even if they are geographically close. Our study cannot determine whether the differences are due to adaptation or drift. Still, wing shape potential could have performance effects on dispersal and reproduction, as the wings are used in search of food, nest, or mating, as well as the potential for dispersal to new areas or the adaptation to more complex landscapes (52–55). Regardless of the evolutionary origin of these differences, the information provided by the GMM analysis can help identify the origin of an intercepted or invasive specimen or separate well-differentiated groups based on characteristics other than the coloration patterns used previously for the different subspecies of *V. orientalis*.

This study also shows that Oriental hornet distributions in northern Africa, the Mediterranean portion of Europe, and the Middle East differ in their forewing shape, especially in the African group, but have overlapping climatic niches. The shapes of the wings of these morphogroups provide a mechanism for identifying the source population of invasive hornets captured outside their natural range. The climatic niche of the African morphogroup is wholly contained within the climate space of Middle Eastern morphogroups (**Figure 6**). Still, the Mediterranean and African climatic niches are different from each other, and the Mediterranean morphogroup is associated with climates outside those related to the Middle Eastern morphogroup. The overlap between the climatic niches of the three morph groups suggests that any of them could become invasive in other parts of the world that share these climate ranges. Furthermore, African-sourced invasive species could be viable in any location where Middle Eastern invasive species may survive, and Mediterranean invasive species could establish ranges where Middle Eastern and African ones cannot.

There have been multiple studies modeling the potential ecological niche of invasive hornets around the world and their potential impacts on native species and natural ecosystems of the invaded areas, including Moo-Llanes (56), Nuñez-Penichet et al. (57), and Werenkraut et al. (19). Our work, although similar to the one by Werenkraut et al. (19), is novel in the fact that it uses different morphogroups and shows how the potential distribution of a species also depends on the particular group introduced as much as it depends on its genetic differences such as the ones observed with specific populations. The use of geometric morphometrics (GMM) has proven to be beneficial in the identification of and characterization of the variability among different, closely related species of insects (58–62) and particularly of hornets (29, 63), much so of those considered incipient or recently introduced species, the ones that are part of a species complex where differences among species in the group are not easily seen or for different variations among the same species.

In this study, we demonstrated that GMM is a valuable tool for separating morphogroups (and possibly population) within a species and how shape differences in morphogroups are related to their geographical distributions. Even with relatively small sample sizes, the significance of our Procrustes ANOVA demonstrates that forewing shape differentiation is detectable in the northern African population. The high proportion of correct classification indicates that smaller but consistent differentiation exists between the Middle Eastern and north Mediterranean populations. Combined with the differences detected in their suitable climatic niches, these results suggest that regional differentiation exists in *V. orientalis*, perhaps driven by biogeographic semi-isolation associated with differences in habitat and the presence of geographic barriers that are not easily visible but can be detected by multivariate shape analysis. That this differentiation is comparatively small is perhaps no surprise since there is also an overlap in climatic niches among the different morphogroups that explains in part what has been observed by other workers and due to the similarity in some of the variables used in the study of ecological niches; some of these works include Ebrahimi and Carpenter (64) and Lioy et al. (65).

Any of the three morphogroups could become invasive in Chile if our niche models are adequate predictors because broad areas of that country fall within the climatic niches. However, the Mediterranean and African morphogroups are amenable to different parts of Chile. Similarly, the three groups can potentially become invasive in the USA based on their climatic niches, and the Mediterranean and African source morphogroups are amenable to different parts of the USA. Forewing morphometrics has good potential for identifying the source of intercepted invasive species. It is possible, of course, that the morphology of an introduced or invasive species could change from its source population because of founder effects, intense local selection in the new location, or even the slower accumulation of drift due to lack of gene flow with the parent population (e.g., 66, 67), and this is the case with at least some introduced insects such as *Diabrotica virgifera* (Coleoptera: Chrysomelidae) in Europe (68–70). While it would be an unusual circumstance for an introduced population to take on a new morphology that is like a natural population other than its parent, it is possible that it could take on a form that is different from all others, in which case a phylogenetic molecular approach would be needed on top of morphometrics to determine the source.

This study also shows that a better understanding of the morphological variation among populations of highly variable species can considerably change our understanding of their potential invasiveness.

## Data Availability

The original contributions presented in the study are included in the article/**Supplementary Material**. Further inquiries can be directed to the corresponding author.
