# Supplementary material for: The Oriental hornet, Vespa orientalis Linnaeus, 1771 (Hymenoptera, Vespidae): diagnosis, potential distribution, and geometric morphometrics across its natural distribution range
Source: Front Insect Sci. 2024 Oct 29;4:1384598. doi: 10.3389/finsc.2024.1384598 (PMC11555395; doi:10.3389/finsc.2024.1384598)
Supplement: Supplementary file 1 [file Table1.docx]

**Table 1.** List of the specimens used for the morphometric analysis (with their corresponding codes) as well as their distribution and the morph-group/ population it tentatively belongs to (all museum specimens, most of them only with the country as distribution information, population/morph-group according to preliminary analysis for all specimens available: MEDI (= the Mediterranean -including the interior of western Europe were it is introduced), AFRI (African: especially from areas around the Sahara desert and excluding the Arabian Peninsula) and MEAS (Middle East- including parts of western Asia).

| **Specimen/ code:** | **Distribution** | **Population/ Shape Group (GMM)** |
| --- | --- | --- |
|  |  |  |
| Perr-0000 | Sahara (no country) | AFRI |
|  |  |  |
| Perr-0001 | Turkey | MEDI |
|  |  |  |
| Perr-0004 | Malta | MEDI |
|  |  |  |
| Perr-0005 | Malta | MEDI |
|  |  |  |
| Perr-0006 | Iraq | MEAS |
|  |  |  |
| Perr-0007 | Iran | MEAS |
|  |  |  |
| Perr-0008 | Italy: Sicily | MEDI |
|  |  |  |
| Perr-0009 | Sahara- central (no country) | AFRI |
|  |  |  |
| Perr-0011 | Croatia-Cilice | MEDI |
|  |  |  |
| Perr-0012 | Croatia-Cilice | MEDI |
|  |  |  |
| Perr-0013 | Egypt | AFRI |
|  |  |  |
| Perr-0014 | Chad | AFRI |
|  |  |  |
| Perr-0016 | Iran | MEAS |
|  |  |  |
| Perr-0017 | Iran | MEAS |
|  |  |  |
| Perr-0018 | Italy: Catania | MEDI |
|  |  |  |
| Perr-0019 | Cyprus | MEDI |
|  |  |  |
| Perr-0020 | Cyprus | MEDI |
|  |  |  |
| Perr-0021 | Cyprus | MEDI |
|  |  |  |
| Perr-0022 | Greece | MEDI |
|  |  |  |
| Perr-0023 | Iran | MEAS |
|  |  |  |
| Perr-0024 | Chad | AFRI |
|  |  |  |
| Perr-0025 | Algeria | AFRI |
|  |  |  |
| Perr-0026 | Oman | MEAS |
|  |  |  |
| Perr-0027 | Oman | MEAS |
|  |  |  |
| Perr-0028 | Oman | MEAS |
|  |  |  |
| Perr-0029 | Egypt | AFRI |
|  |  |  |
| V_orient_Nsubsp_Tur_004 | Turkey | MEAS |
|  |  |  |
| V_orient_Nsubsp_Isr_005 | Israel | MEDI |
|  |  |  |
| V_orient_Nsubsp_UAE_001 | United Arab Emirates | MEAS |
|  |  |  |
| V_orient_Nsubsp_UAE_002 | United Arab Emirates | MEAS |
|  |  |  |
| V_orient_Nsubsp_UAE_003 | United Arab Emirates | MEAS |
|  |  |  |

* **Name codes:** *Perr*- for specimens from Perrard et al (2014) and the AMNH, *V_orient_* for specimens from the BMEC.
