# Supplementary material for: The Oriental hornet, Vespa orientalis Linnaeus, 1771 (Hymenoptera, Vespidae): diagnosis, potential distribution, and geometric morphometrics across its natural distribution range
Source: Front Insect Sci. 2024 Oct 29;4:1384598. doi: 10.3389/finsc.2024.1384598 (PMC11555395; doi:10.3389/finsc.2024.1384598)
Supplement: Supplementary file 2 [file Table2.docx]

LM=19

660.00000 1631.00000

1420.00000 1640.00000

1522.00000 1707.00000

1934.00000 1655.00000

1948.00000 1445.00000

2038.00000 1518.00000

2118.00000 1487.00000

2380.00000 1431.00000

2497.00000 1423.00000

2356.00000 1227.00000

2221.00000 1267.00000

2028.00000 1312.00000

1789.00000 1119.00000

1333.00000 1292.00000

1241.00000 1208.00000

683.00000 1552.00000

690.00000 1589.00000

1424.00000 1418.00000

1956.00000 1380.00000

ID=AFRI-Perr-0000

LM=19

737.00000 1694.00000

1423.00000 1758.00000

1530.00000 1834.00000

1896.00000 1824.00000

1946.00000 1609.00000

2014.00000 1696.00000

2091.00000 1664.00000

2305.00000 1638.00000

2415.00000 1635.00000

2307.00000 1441.00000

2192.00000 1468.00000

2028.00000 1489.00000

1813.00000 1298.00000

1386.00000 1436.00000

1307.00000 1349.00000

770.00000 1617.00000

770.00000 1652.00000

1464.00000 1553.00000

1951.00000 1549.00000

ID=MEDI-Perr-0001

LM=19

625.00000 1446.00000

1555.00000 1484.00000

1677.00000 1567.00000

2162.00000 1525.00000

2199.00000 1247.00000

2302.00000 1355.00000

2416.00000 1305.00000

2692.00000 1244.00000

2867.00000 1230.00000

2687.00000 1013.00000

2527.00000 1056.00000

2313.00000 1097.00000

1990.00000 864.00000

1452.00000 1060.00000

1349.00000 944.00000

676.00000 1331.00000

677.00000 1385.00000

1559.00000 1210.00000

2210.00000 1185.00000

ID=MEDI-Perr-0004

LM=19

686.00000 1261.00000

1607.00000 1282.00000

1749.00000 1368.00000

2217.00000 1319.00000

2247.00000 1043.00000

2363.00000 1141.00000

2470.00000 1092.00000

2732.00000 1031.00000

2904.00000 1014.00000

2729.00000 800.00000

2567.00000 849.00000

2359.00000 894.00000

2029.00000 668.00000

1502.00000 870.00000

1394.00000 757.00000

738.00000 1150.00000

740.00000 1197.00000

1612.00000 1014.00000

2259.00000 982.00000

ID=MEDI-Perr-0005

LM=19

849.00000 1847.00000

1504.00000 1911.00000

1585.00000 1976.00000

1960.00000 1968.00000

1981.00000 1770.00000

2055.00000 1849.00000

2129.00000 1825.00000

2337.00000 1804.00000

2435.00000 1810.00000

2331.00000 1613.00000

2230.00000 1635.00000

2069.00000 1654.00000

1856.00000 1479.00000

1459.00000 1600.00000

1385.00000 1518.00000

869.00000 1780.00000

875.00000 1811.00000

1533.00000 1711.00000

1987.00000 1715.00000

ID=MEAS-Perr-0006

LM=19

411.00000 1349.00000

1250.00000 1305.00000

1379.00000 1381.00000

1844.00000 1294.00000

1838.00000 1046.00000

1941.00000 1136.00000

2035.00000 1087.00000

2294.00000 1010.00000

2433.00000 992.00000

2275.00000 776.00000

2125.00000 830.00000

1927.00000 890.00000

1645.00000 706.00000

1151.00000 927.00000

1047.00000 835.00000

446.00000 1248.00000

455.00000 1287.00000

1260.00000 1051.00000

1843.00000 983.00000

ID=MEAS-Perr-0007

LM=19

1052.00000 1543.00000

1793.00000 1509.00000

1898.00000 1571.00000

2283.00000 1510.00000

2292.00000 1280.00000

2386.00000 1354.00000

2465.00000 1306.00000

2696.00000 1237.00000

2829.00000 1225.00000

2659.00000 1061.00000

2502.00000 1119.00000

2377.00000 1150.00000

2118.00000 978.00000

1692.00000 1183.00000

1597.00000 1104.00000

1077.00000 1454.00000

1081.00000 1495.00000

1788.00000 1289.00000

2296.00000 1222.00000

ID=MEDI-Perr-0008

LM=19

988.00000 1557.00000

1661.00000 1537.00000

1754.00000 1594.00000

2093.00000 1542.00000

2097.00000 1344.00000

2176.00000 1416.00000

2237.00000 1389.00000

2469.00000 1336.00000

2575.00000 1323.00000

2443.00000 1161.00000

2316.00000 1195.00000

2156.00000 1237.00000

1938.00000 1086.00000

1553.00000 1245.00000

1473.00000 1174.00000

1017.00000 1482.00000

1024.00000 1512.00000

1630.00000 1346.00000

2102.00000 1291.00000

ID=AFRI-Perr-0009

LM=19

1002.00000 1589.00000

1658.00000 1569.00000

1757.00000 1633.00000

2138.00000 1577.00000

2150.00000 1365.00000

2227.00000 1442.00000

2303.00000 1403.00000

2518.00000 1350.00000

2621.00000 1335.00000

2486.00000 1155.00000

2378.00000 1195.00000

2215.00000 1242.00000

1979.00000 1078.00000

1584.00000 1257.00000

1510.00000 1177.00000

1024.00000 1510.00000

1036.00000 1540.00000

1672.00000 1363.00000

2155.00000 1304.00000

ID=MEDI-Perr-0011

LM=19

448.00000 1619.00000

1148.00000 1597.00000

1253.00000 1659.00000

1620.00000 1601.00000

1637.00000 1393.00000

1718.00000 1467.00000

1794.00000 1433.00000

2014.00000 1381.00000

2111.00000 1377.00000

1986.00000 1186.00000

1874.00000 1226.00000

1712.00000 1270.00000

1478.00000 1100.00000

1073.00000 1289.00000

982.00000 1210.00000

471.00000 1542.00000

477.00000 1574.00000

1161.00000 1391.00000

1636.00000 1338.00000

ID=MEDI-Perr-0012

LM=19

368.00000 1459.00000

1075.00000 1470.00000

1180.00000 1535.00000

1572.00000 1499.00000

1569.00000 1300.00000

1658.00000 1372.00000

1746.00000 1335.00000

1987.00000 1276.00000

2104.00000 1270.00000

1967.00000 1089.00000

1838.00000 1126.00000

1674.00000 1164.00000

1439.00000 980.00000

1013.00000 1146.00000

928.00000 1063.00000

392.00000 1380.00000

397.00000 1416.00000

1086.00000 1262.00000

1577.00000 1241.00000

ID=AFRI-Perr-0013

LM=19

404.00000 1289.00000

1294.00000 1419.00000

1403.00000 1515.00000

1875.00000 1530.00000

1920.00000 1284.00000

2019.00000 1385.00000

2109.00000 1364.00000

2402.00000 1344.00000

2554.00000 1348.00000

2428.00000 1101.00000

2256.00000 1123.00000

2050.00000 1141.00000

1802.00000 889.00000

1240.00000 1009.00000

1144.00000 897.00000

444.00000 1194.00000

449.00000 1242.00000

1327.00000 1163.00000

1939.00000 1216.00000

ID=AFRI-Perr-0014

LM=19

485.00000 1521.00000

1098.00000 1565.00000

1194.00000 1631.00000

1539.00000 1609.00000

1563.00000 1411.00000

1637.00000 1482.00000

1714.00000 1451.00000

1908.00000 1415.00000

2012.00000 1406.00000

1885.00000 1244.00000

1790.00000 1270.00000

1640.00000 1299.00000

1421.00000 1137.00000

1062.00000 1263.00000

994.00000 1186.00000

516.00000 1447.00000

520.00000 1479.00000

1122.00000 1369.00000

1570.00000 1360.00000

ID=MEAS-Perr-0016

LM=19

454.00000 1333.00000

1077.00000 1427.00000

1154.00000 1498.00000

1516.00000 1508.00000

1562.00000 1319.00000

1622.00000 1396.00000

1683.00000 1377.00000

1880.00000 1367.00000

1985.00000 1368.00000

1896.00000 1186.00000

1778.00000 1207.00000

1638.00000 1222.00000

1464.00000 1037.00000

1068.00000 1132.00000

1002.00000 1046.00000

481.00000 1263.00000

484.00000 1299.00000

1124.00000 1242.00000

1574.00000 1269.00000

ID=MEAS-Perr-0017

LM=19

590.00000 1369.00000

1342.00000 1400.00000

1449.00000 1477.00000

1836.00000 1433.00000

1865.00000 1209.00000

1951.00000 1294.00000

2037.00000 1257.00000

2262.00000 1210.00000

2398.00000 1203.00000

2262.00000 1024.00000

2130.00000 1053.00000

1963.00000 1083.00000

1706.00000 891.00000

1263.00000 1060.00000

1189.00000 967.00000

630.00000 1281.00000

632.00000 1321.00000

1352.00000 1177.00000

1874.00000 1152.00000

ID=MEDI-Perr-0018

LM=19

391.00000 1508.00000

1079.00000 1613.00000

1176.00000 1694.00000

1539.00000 1708.00000

1585.00000 1502.00000

1651.00000 1592.00000

1733.00000 1566.00000

1975.00000 1549.00000

2067.00000 1559.00000

1972.00000 1355.00000

1847.00000 1378.00000

1689.00000 1389.00000

1477.00000 1183.00000

1064.00000 1287.00000

992.00000 1198.00000

419.00000 1438.00000

416.00000 1475.00000

1134.00000 1410.00000

1599.00000 1444.00000

ID=MEDI-Perr-0019

LM=19

181.00000 1760.00000

877.00000 1933.00000

957.00000 2017.00000

1342.00000 2068.00000

1416.00000 1860.00000

1475.00000 1955.00000

1552.00000 1939.00000

1788.00000 1945.00000

1892.00000 1963.00000

1821.00000 1745.00000

1692.00000 1761.00000

1525.00000 1762.00000

1338.00000 1529.00000

876.00000 1599.00000

814.00000 1501.00000

217.00000 1689.00000

218.00000 1726.00000

938.00000 1727.00000

1434.00000 1810.00000

ID=MEDI-Perr-0020

LM=19

227.00000 1659.00000

892.00000 1780.00000

977.00000 1858.00000

1335.00000 1869.00000

1389.00000 1670.00000

1449.00000 1758.00000

1524.00000 1737.00000

1743.00000 1725.00000

1844.00000 1739.00000

1757.00000 1541.00000

1646.00000 1556.00000

1490.00000 1562.00000

1311.00000 1364.00000

876.00000 1461.00000

815.00000 1374.00000

264.00000 1589.00000

263.00000 1623.00000

939.00000 1580.00000

1401.00000 1617.00000

ID=MEDI-Perr-0021

LM=19

760.00000 1532.00000

1448.00000 1531.00000

1552.00000 1596.00000

1934.00000 1533.00000

1940.00000 1322.00000

2025.00000 1401.00000

2106.00000 1359.00000

2322.00000 1301.00000

2441.00000 1287.00000

2299.00000 1121.00000

2179.00000 1160.00000

2015.00000 1198.00000

1784.00000 1033.00000

1360.00000 1217.00000

1286.00000 1133.00000

787.00000 1453.00000

789.00000 1489.00000

1444.00000 1325.00000

1947.00000 1264.00000

ID=MEDI-Perr-0022

LM=19

744.00000 1467.00000

1572.00000 1533.00000

1692.00000 1626.00000

2163.00000 1603.00000

2213.00000 1357.00000

2300.00000 1460.00000

2396.00000 1429.00000

2663.00000 1394.00000

2808.00000 1398.00000

2689.00000 1152.00000

2537.00000 1183.00000

2323.00000 1214.00000

2060.00000 970.00000

1533.00000 1132.00000

1435.00000 1014.00000

779.00000 1365.00000

789.00000 1413.00000

1636.00000 1273.00000

2229.00000 1288.00000

ID=MEAS-Perr-0023

LM=19

750.00000 1297.00000

1510.00000 1344.00000

1635.00000 1419.00000

1997.00000 1387.00000

2010.00000 1174.00000

2104.00000 1259.00000

2188.00000 1227.00000

2442.00000 1182.00000

2568.00000 1168.00000

2422.00000 981.00000

2272.00000 1017.00000

2101.00000 1053.00000

1865.00000 855.00000

1422.00000 999.00000

1343.00000 911.00000

792.00000 1207.00000

791.00000 1250.00000

1506.00000 1123.00000

2014.00000 1123.00000

ID=AFRI-Perr-0024

LM=19

387.00000 1065.00000

1052.00000 1047.00000

1167.00000 1111.00000

1533.00000 1046.00000

1534.00000 850.00000

1623.00000 920.00000

1691.00000 886.00000

1931.00000 820.00000

2037.00000 810.00000

1895.00000 628.00000

1764.00000 678.00000

1612.00000 723.00000

1388.00000 543.00000

976.00000 734.00000

884.00000 659.00000

401.00000 992.00000

412.00000 1023.00000

1057.00000 843.00000

1540.00000 792.00000

ID=AFRI-Perr-0025

LM=19

985.00000 1567.00000

1689.00000 1581.00000

1807.00000 1651.00000

2172.00000 1621.00000

2211.00000 1406.00000

2291.00000 1486.00000

2365.00000 1457.00000

2589.00000 1418.00000

2686.00000 1415.00000

2586.00000 1213.00000

2460.00000 1250.00000

2298.00000 1281.00000

2051.00000 1112.00000

1641.00000 1261.00000

1560.00000 1183.00000

1020.00000 1490.00000

1020.00000 1523.00000

1722.00000 1369.00000

2216.00000 1348.00000

ID=MEAS-Perr-0026

LM=19

867.00000 1418.00000

1657.00000 1423.00000

1775.00000 1497.00000

2199.00000 1453.00000

2223.00000 1216.00000

2314.00000 1303.00000

2405.00000 1262.00000

2656.00000 1207.00000

2774.00000 1203.00000

2645.00000 979.00000

2497.00000 1030.00000

2313.00000 1072.00000

2022.00000 888.00000

1578.00000 1061.00000

1481.00000 972.00000

905.00000 1328.00000

907.00000 1369.00000

1674.00000 1187.00000

2228.00000 1150.00000

ID=MEAS-Perr-0027

LM=19

1004.00000 1617.00000

1709.00000 1565.00000

1840.00000 1627.00000

2201.00000 1543.00000

2193.00000 1337.00000

2287.00000 1409.00000

2363.00000 1365.00000

2587.00000 1299.00000

2702.00000 1277.00000

2561.00000 1098.00000

2437.00000 1149.00000

2281.00000 1197.00000

2026.00000 1049.00000

1623.00000 1246.00000

1544.00000 1168.00000

1028.00000 1540.00000

1032.00000 1572.00000

1715.00000 1350.00000

2194.00000 1281.00000

ID=MEAS-Perr-0028

LM=19

704.00000 1018.00000

1533.00000 1014.00000

1647.00000 1084.00000

2084.00000 1022.00000

2093.00000 796.00000

2188.00000 880.00000

2296.00000 833.00000

2566.00000 757.00000

2700.00000 744.00000

2545.00000 537.00000

2390.00000 586.00000

2194.00000 643.00000

1931.00000 442.00000

1447.00000 642.00000

1345.00000 546.00000

727.00000 929.00000

735.00000 972.00000

1529.00000 772.00000

2103.00000 731.00000

ID=AFRI-Perr-0029

LM=19

744.00000 632.00000

1779.00000 974.00000

1902.00000 1105.00000

2474.00000 1222.00000

2603.00000 920.00000

2691.00000 1076.00000

2843.00000 1054.00000

3164.00000 1066.00000

3355.00000 1109.00000

3251.00000 800.00000

3041.00000 793.00000

2787.00000 777.00000

2558.00000 416.00000

1822.00000 480.00000

1738.00000 316.00000

816.00000 525.00000

812.00000 590.00000

1914.00000 675.00000

2640.00000 843.00000

ID=MEAS_005

LM=19

827.00000 692.00000

1768.00000 839.00000

1880.00000 937.00000

2367.00000 962.00000

2445.00000 687.00000

2533.00000 808.00000

2648.00000 774.00000

2939.00000 750.00000

3092.00000 748.00000

2962.00000 493.00000

2806.00000 522.00000

2568.00000 542.00000

2268.00000 288.00000

1715.00000 414.00000

1627.00000 303.00000

888.00000 596.00000

883.00000 643.00000

1811.00000 580.00000

2463.00000 613.00000

ID=MEDI_004

LM=19

792.00000 687.00000

1820.00000 944.00000

1954.00000 1074.00000

2485.00000 1151.00000

2583.00000 849.00000

2676.00000 1003.00000

2822.00000 974.00000

3107.00000 968.00000

3285.00000 994.00000

3164.00000 696.00000

2982.00000 709.00000

2740.00000 713.00000

2457.00000 391.00000

1811.00000 463.00000

1738.00000 327.00000

872.00000 581.00000

868.00000 637.00000

1887.00000 659.00000

2609.00000 776.00000

ID=MEAS_001

LM=19

814.00000 688.00000

1849.00000 933.00000

1999.00000 1065.00000

2527.00000 1143.00000

2608.00000 842.00000

2701.00000 986.00000

2838.00000 959.00000

3152.00000 961.00000

3324.00000 972.00000

3196.00000 679.00000

3002.00000 698.00000

2773.00000 700.00000

2480.00000 385.00000

1828.00000 467.00000

1754.00000 327.00000

893.00000 582.00000

891.00000 639.00000

1913.00000 658.00000

2637.00000 768.00000

ID=MEAS_002

LM=19

872.00000 689.00000

1886.00000 904.00000

2045.00000 1029.00000

2567.00000 1089.00000

2660.00000 786.00000

2756.00000 923.00000

2881.00000 893.00000

3193.00000 876.00000

3382.00000 889.00000

3238.00000 596.00000

3057.00000 624.00000

2812.00000 641.00000

2496.00000 336.00000

1866.00000 441.00000

1786.00000 306.00000

941.00000 581.00000

946.00000 631.00000

1956.00000 622.00000

2673.00000 708.00000

ID=MEAS_003
