# Supplementary material for: The Oriental hornet, Vespa orientalis Linnaeus, 1771 (Hymenoptera, Vespidae): diagnosis, potential distribution, and geometric morphometrics across its natural distribution range
Source: Front Insect Sci. 2024 Oct 29;4:1384598. doi: 10.3389/finsc.2024.1384598 (PMC11555395; doi:10.3389/finsc.2024.1384598)
Supplement: Supplementary file 3 [file Table3.docx]

**Supplement 3.** Procrustes analysis for specimens used for the geometric morphometrics analysis of the hind wings of specimens of *V. orientalis*.

**Average shape:**

**Landmark. Axis 1 (x) Axis 2 (y)**

1 -0.41356071 0.02990375

2 -0.11840789 0.06699706

3 -0.07746079 0.09952135

4 0.08144187 0.09970772

5 0.09867915 0.01231153

6 0.12966585 0.04894073

7 0.16450527 0.03777776

8 0.25989546 0.02755677

9 0.30850298 0.02895276

10 0.26297695 -0.05362916

11 0.20905910 -0.04349497

12 0.13930825 -0.03551959

13 0.04870063 -0.11948105

14 -0.13378394 -0.07067129

15 -0.16396822 -0.10881685

16 -0.39799816 -0.00229042

17 -0.39743815 0.01302506

18 -0.10389802 -0.01996299

19 0.10378036 -0.01082816

**Procrustes sums of squares:** 0.008553706349618067

**Tangent sums of squares:** 0.008550931574244918
