# Supplementary material for: The Oriental hornet, Vespa orientalis Linnaeus, 1771 (Hymenoptera, Vespidae): diagnosis, potential distribution, and geometric morphometrics across its natural distribution range
Source: Front Insect Sci. 2024 Oct 29;4:1384598. doi: 10.3389/finsc.2024.1384598 (PMC11555395; doi:10.3389/finsc.2024.1384598)

**Supplement 4.** Outliners for the results of the procrustes analysis of hind wing landmarks of specimens of *V. orientalis* used in this study.


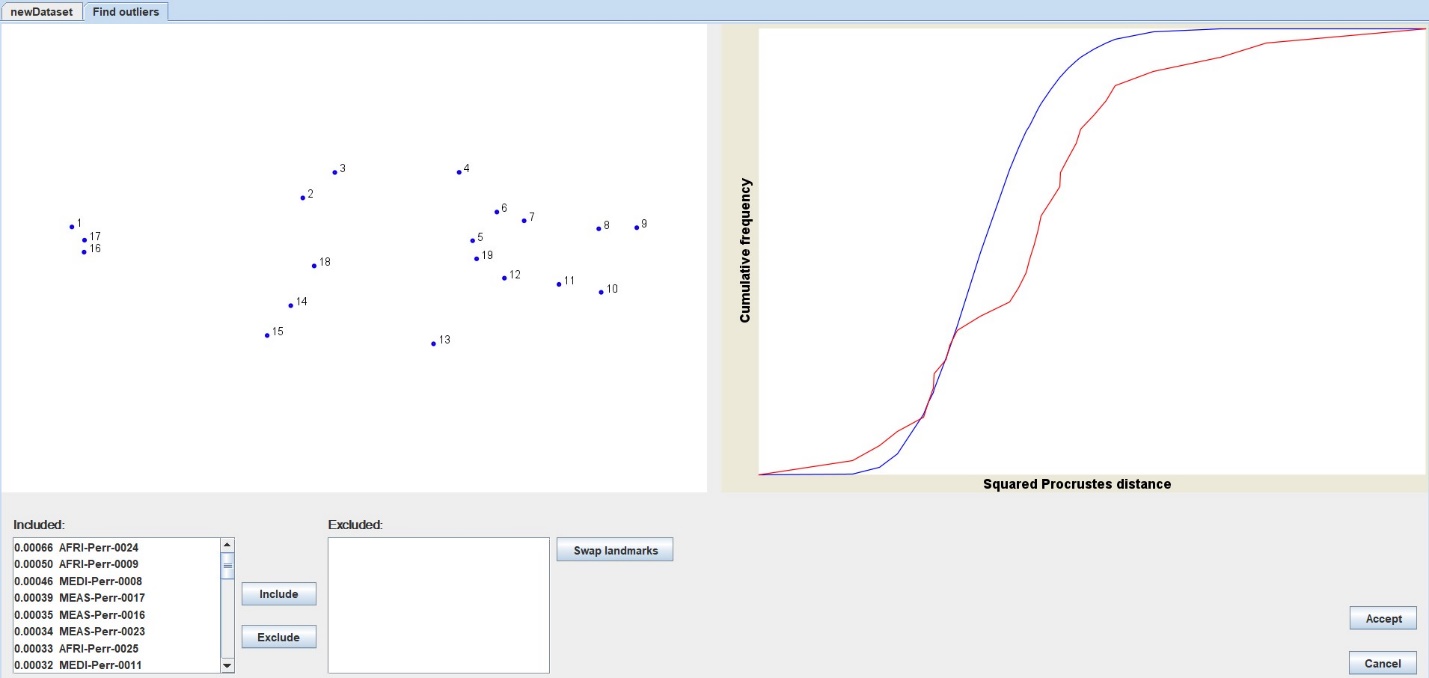

Supplement: Supplementary file 4 [file Table4.docx]
