# Supplementary material for: The Oriental hornet, Vespa orientalis Linnaeus, 1771 (Hymenoptera, Vespidae): diagnosis, potential distribution, and geometric morphometrics across its natural distribution range
Source: Front Insect Sci. 2024 Oct 29;4:1384598. doi: 10.3389/finsc.2024.1384598 (PMC11555395; doi:10.3389/finsc.2024.1384598)
Supplement: Supplementary file 5 [file Table5.docx]

**Supplement 5.** Results of the Principal Component Analysis (PCA) for the shape variation of the hind wing of specimens of V. orientalis.

**PC Eigenvalues % Variance Cumulative %**

1. 0.00007995 28.049 28.049

2. 0.00004598 16.132 44.181

3. 0.00003443 12.078 56.260

4. 0.00002872 10.077 66.336

5. 0.00002051 7.195 73.531

6. 0.00001663 5.834 79.365

7. 0.00001114 3.907 83.273

8. 0.00000910 3.191 86.464

9. 0.00000755 2.649 89.113

10. 0.00000659 2.311 91.424

11. 0.00000500 1.755 93.179

12. 0.00000391 1.372 94.551

13. 0.00000308 1.080 95.631

14. 0.00000231 0.812 96.443

15. 0.00000219 0.767 97.210

16. 0.00000160 0.562 97.772

17. 0.00000141 0.494 98.266

18. 0.00000105 0.370 98.636

19. 0.00000090 0.317 98.953

20. 0.00000077 0.269 99.222

21. 0.00000058 0.202 99.425

22. 0.00000044 0.153 99.578

23. 0.00000033 0.117 99.694

24. 0.00000028 0.098 99.792

25. 0.00000022 0.077 99.869

26. 0.00000017 0.059 99.928

27. 0.00000013 0.045 99.973

28. 0.00000005 0.018 99.991

29. 0.00000002 0.006 99.997

30. 0.00000001 0.003 100.000

**Total variance:** 0.00028503

**Variance of the eigenvalues:** 0.0000000002706

**Eigenvalue variance scaled by total variance:** 0.00333

**Eigenvalue variance scaled by total variance and number of variables:** 0.11666
