# Supplementary material for: The Oriental hornet, Vespa orientalis Linnaeus, 1771 (Hymenoptera, Vespidae): diagnosis, potential distribution, and geometric morphometrics across its natural distribution range
Source: Front Insect Sci. 2024 Oct 29;4:1384598. doi: 10.3389/finsc.2024.1384598 (PMC11555395; doi:10.3389/finsc.2024.1384598)
Supplement: Supplementary file 6 [file Table6.docx]

**Supplement 6.** Canonical Variate Analysis (CVA) of specimens of *V. orientalis* belonging to the three populations confirmed by the PCA.

**Classification criterion:** **Region/ Population**

**Observations:**

1. AFRI 7

2. MEAS 12

3. MEDI 12

**Variation among groups, scaled by the inverse of the within-group variation:**

**Eigenvalues % Variance Cumulative %**

1. 84.42664317 88.536 88.536

2. 10.93219026 11.464 100.000

**Mahalanobis distances among groups:**

**AFRI** **MEAS**

**MEAS** 21.0824 _____

**MEDI** 21.2923 7.1463

**P-values from permutation tests (10000 permutation rounds) for Mahalanobis distances among groups:**

**AFRI** **MEAS**

**MEAS** <.0001 _____

**MEDI** <.0001 <.0001

**Procrustes distances among groups:**

**AFRI** **MEAS**

**MEAS** 0.0166 _____

**MEDI** 0.0150 0.0065

**P-values from permutation tests (10000 permutation rounds) for Procrustes distances among groups:**

**AFRI** **MEAS**

**MEAS** <.0001 _____

MEDI 0.0003 0.4026

**Canonical coefficients:**

**CV1** **CV2**

x1 -491.5358 927.5151

y1 -2368.1057 2468.2065

x2 1052.8825 720.9785

y2 -3175.7131 -44.9713

x3 -573.5810 80.1628

y3 1316.6553 492.8947

x4 -727.3043 5.7383

y4 530.5429 80.2129

x5 1726.2204 -932.7081

y5 1177.0008 650.8115

x6 896.6786 -1405.6277

y6 -54.5934 -575.3125

x7 -589.3511 571.3258

y7 -5.8957 -1028.4019

x8 514.8189 -390.6290

y8 218.9607 999.9258

x9 345.1741 125.8787

y9 -658.2972 -197.5851

x10 -312.9879 87.2295

y10 820.3795 -1047.8488

x11 306.4583 616.2122

y11 -1687.1997 1897.1650

x12 -48.7811 -410.5281

y12 1030.1072 287.9652

x13 856.3446 -46.5927

y13 485.5768 -1401.3631

x14 604.8776 77.6784

y14 -473.5396 -393.1453

x15 -901.9058 47.6466

y15 -556.8359 1701.0767

x16 518.8437 143.2475

y16 149.2687 -502.0454

x17 537.1691 -1254.7903

y17 3551.5743 -2400.6788

x18 -1194.9342 -221.0310

y18 -323.6629 -387.5304

x19 -2519.0868 1258.2934

y19 23.7770 -599.3757
