# Supplementary material for: The Oriental hornet, Vespa orientalis Linnaeus, 1771 (Hymenoptera, Vespidae): diagnosis, potential distribution, and geometric morphometrics across its natural distribution range
Source: Front Insect Sci. 2024 Oct 29;4:1384598. doi: 10.3389/finsc.2024.1384598 (PMC11555395; doi:10.3389/finsc.2024.1384598)
Supplement: Supplementary file 7 [file Table7.docx]

**Supplement 7.** Discriminant Function Analysis (degree of similarity) of the different morph groups/ populations of *V. orientalis* found in this study.

**Comparison: AFRI -- MEAS**

Difference between means:

Procrustes distance: 0.01660380

Mahalanobis distance: 5.4150

T-square: 129.6352, P-value (parametric): 0.8463

P-values for permutation tests (1000 permutation runs):

Procrustes distance: <.0001

T-square: <.0001

Classification/misclassification tables:

Group 1: AFRI

Group 2: MEAS

From discriminant function:

Group Group 1 Group 2 Total

Group 1 7 0 7

Group 2 0 12 12

From cross-validation:

Group Group 1 Group 2 Total

Group 1 6 1 7

Group 2 1 11 12

**Comparison: AFRI -- MEDI**

Difference between means:

Procrustes distance: 0.01500391

Mahalanobis distance: 8.2005

T-square: 297.3104, P-value (parametric): 0.6620

P-values for permutation tests (1000 permutation runs):

Procrustes distance: 0.0010

T-square: <.0001

Classification/misclassification tables:

Group 1: AFRI

Group 2: MEDI

From discriminant function:

Group Group 1 Group 2 Total

Group 1 7 0 7

Group 2 0 12 12

From cross-validation:

Group Group 1 Group 2 Total

Group 1 7 0 7

Group 2 0 12 12

**Comparison: MEAS -- MEDI**

Difference between means:

Procrustes distance: 0.00650700

Mahalanobis distance: 7.0211

T-square: 295.7724, P-value (parametric): 0.7859

P-values for permutation tests (1000 permutation runs):

Procrustes distance: 0.4040

T-square: 0.0140

Classification/misclassification tables:

Group 1: MEAS

Group 2: MEDI

From discriminant function:

Group Group 1 Group 2 Total

Group 1 12 0 12

Group 2 0 12 12

From cross-validation:

Group Group 1 Group 2 Total

Group 1 5 7 12

Group 2 7 5 12
