# Supplementary material for: The Oriental hornet, Vespa orientalis Linnaeus, 1771 (Hymenoptera, Vespidae): diagnosis, potential distribution, and geometric morphometrics across its natural distribution range
Source: Front Insect Sci. 2024 Oct 29;4:1384598. doi: 10.3389/finsc.2024.1384598 (PMC11555395; doi:10.3389/finsc.2024.1384598)
Supplement: Supplementary file 8 [file Table8.docx]

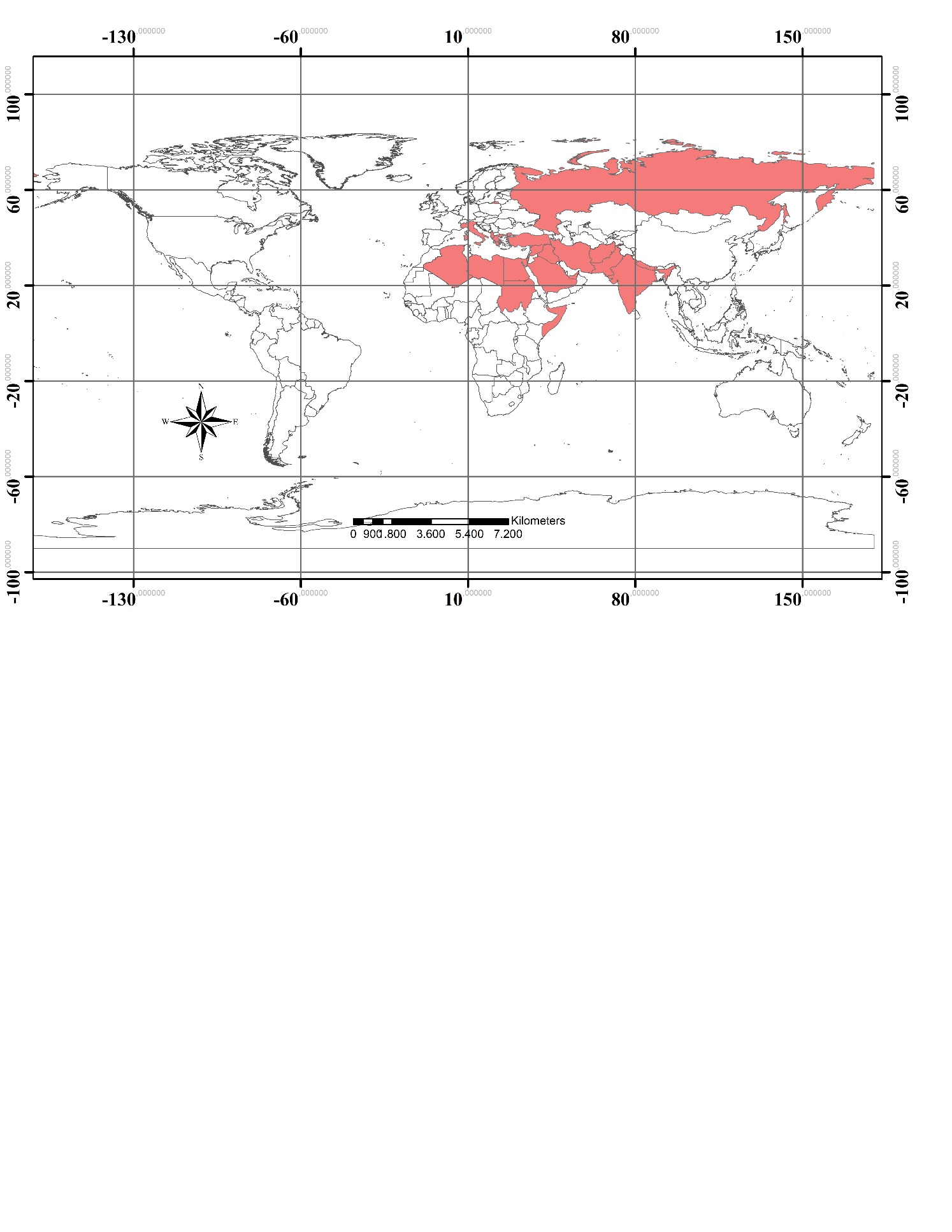


**Supplement 8.** Current known distribution of *V. orientalis*, the coral color indicates the places where the species has been reported.
